# Supplementary material for: 3D Hollow rGO Microsphere Decorated with ZnO Nanoparticles as Efficient Sulfur Host for High-Performance Li-S Battery
Source: Nanomaterials (Basel). 2020 Aug 20;10(9):1633. doi: 10.3390/nano10091633 (PMC7559558; doi:10.3390/nano10091633)
Supplement: Supplementary file 1 [file nanomaterials-10-01633-s001.pdf]

nanomaterials

MDPI

Supporting Information

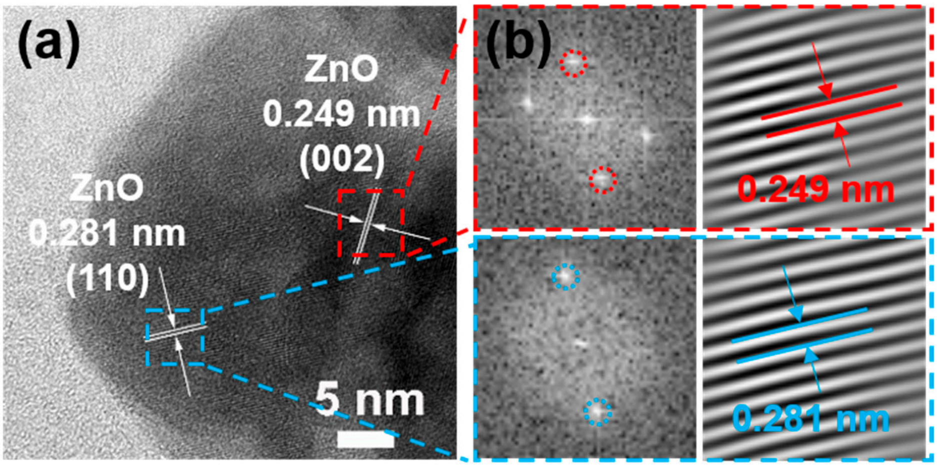

Figure S1. (**a**) HRTEM, (**b**) Fourier transform (FFT) pattern and the inverse FFT crystalline lattice image of 3D-ZnO/rGO.

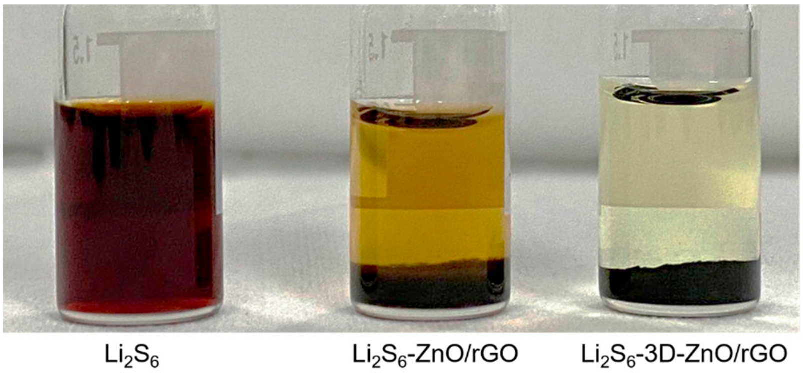

Figure S2. Optical observation of LiPS adsorption by ZnO/rGO and 3D-ZnO/rGO after 24 h.

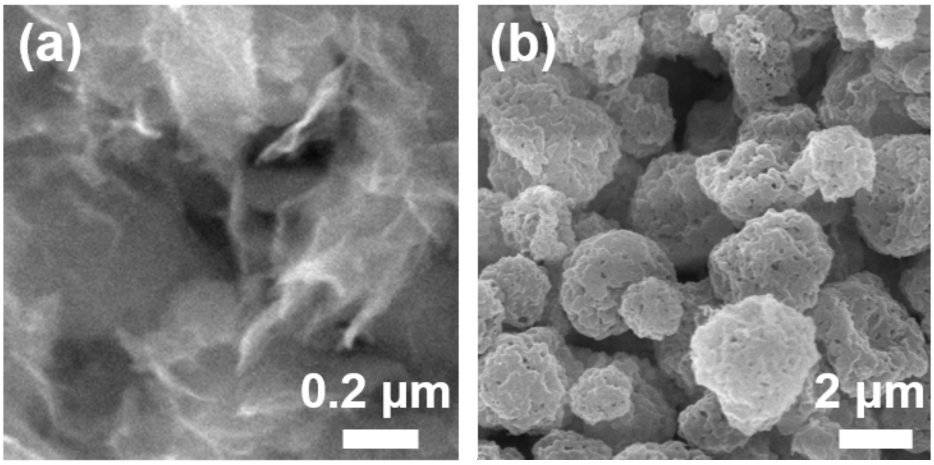

Figure S3. The SEM images of (**a**) S-ZnO/rGO and (**b**) S-3D-ZnO/rGO after 100 cycles at 1 C.

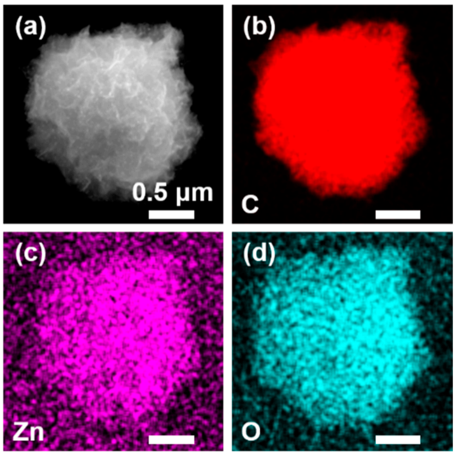

Figure S4. (**a**) SEM image of 3D-ZnO/rGO, and element distribution mapping images for (**b**) C, (**c**) Zn and (**d**) O of 3D-ZnO/rGO.

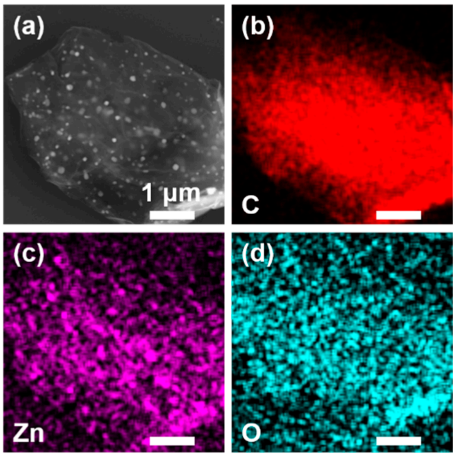

Figure S5. (**a**) SEM image of ZnO/rGO, and element distribution mapping images for (**b**) C, (**c**) Zn and (**d**) O of ZnO/rGO.

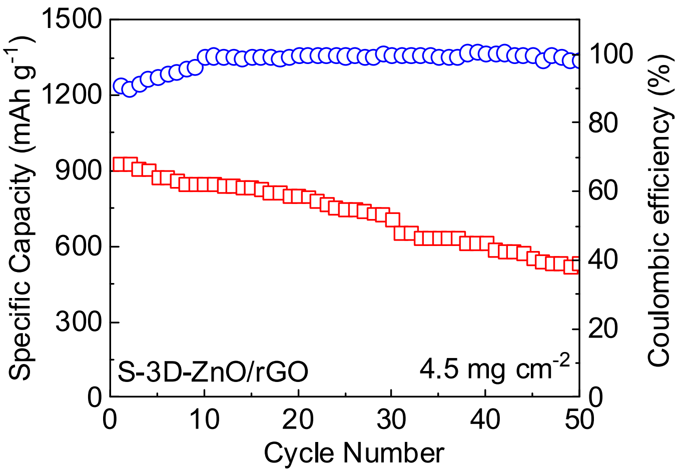

Figure S6. Cycling performance of cell with S-3D-ZnO/rGO at 0.1 C (sulfur loading: 4.5 mg cm^{-2}) (The red curve corresponds to the specific discharge capacity, and the blue curve corresponds to coulombic efficiency).
